# Vitamin D and Musculoskeletal Status in Nova Scotian Women Who Wear Concealing Clothing

**DOI:** 10.3390/nu4050399

**Published:** 2012-05-24

**Authors:** Rani C. I. Ojah, Jo M. Welch

**Affiliations:** School of Health and Human Performance, Dalhousie University, Halifax, NS B3H 3J5, Canada; Email: rani.ojah@dal.ca

**Keywords:** vitamin D, vitamin D deficiency, diet, northern latitude, bone status, muscle strength, hijab, clothing, Muslim, women

## Abstract

Bone and muscle weakness due to vitamin D deficiency is common among Muslim women who reside in sunny, equatorial countries. The purpose of this study was to determine if living in a northern maritime location additionally disadvantages women who wear concealing clothes. A cross-sectional matched pair design was used to compare women who habitually wore concealing clothing with women who dressed according to western norms. Each premenopausal hijab-wearing woman (*n* = 11) was matched by age, height, weight and skin tone with a western-dressed woman. Subjects were tested by hand grip dynamometry to assess muscular strength and by quantitative ultrasound at the calcaneus to assess bone status. Nutritional intake was obtained by 24 h recall. Serum 25-hydroxyvitamin D (s-25(OH)D) status was determined in seven matched pairs. The hijab group had lower s-25(OH)D than women who wore western clothes (40 ± 28 *vs.* 81 ± 32 nmol/L, *p*= 0.01). Grip strength in the right hand was lower in the hijab-wearing women (*p* = 0.05) but this appeared to be due to less participation in intense exercise. Bone status did not differ between groups (*p*= 0.9). Dietary intake of vitamin D was lower in the hijab-wearers (316 ± 353 *vs.* 601 ± 341 IU/day, *p*= 0.001). This pilot study suggests that women living in a northern maritime location appear to be at risk for vitamin D insufficiency and therefore should consider taking vitamin D supplements.

## 1. Introduction

Women who wear concealing clothing and live in northern latitudes may be at greater risk for vitamin D deficiency due to seasonal unavailability of the ultraviolet B radiation (UVB) required for cutaneous conversion of vitamin D. Indeed, women who dress accordingly in northern European countries such as Norway, 60° N [1], Denmark, 56° N [2], and England, 52° N [3] are more prone to vitamin D deficiency than are women who wear western style clothing. However, information regarding the vitamin D status of women who wear concealing clothing and reside in Canadian communities, which mostly lie in latitudes from 42° N to 69° N, has not been reported. Torontonians of South Asian ancestry had s-25(OH)D levels about half that of Caucasians but no information about clothing was included in that study's analysis [4] or in an earlier multiethnic vitamin D study in Toronto [5]. Given that Canadian women who wear western style clothing in such disparate locations of the country as Inuvik, 68° N [6], Calgary, 51° N [7], Toronto, 43° N [8] and Newfoundland, 46° N [9] often have insufficient circulating levels of 25(OH)D, the likelihood of even lower levels of s-25(OH)D in covered women was suspected. 

Inadequate vitamin D status can affect bone integrity thereby predisposing adults to osteoporosis [10], and bone pain [11]. Girls who grow up in conservative Islamic countries tend to be vitamin D deficient and have poor bone status [12,13,14], which can affect their capacity to reach their peak bone mass potential [15]. A deficiency of vitamin D can also adversely affect muscle function although the mechanism by which this occurs is not yet clear [16]. Bone and muscle pain reported by young immigrant Muslim women living in Switzerland was typically misattributed to other conditions [17] but was resolved with vitamin D therapy [18]. Hypovitaminosis D may also decrease muscular strength in children [15] and adults [19] and therefore impact quality of life. 

This study investigated if Nova Scotian women who wear full body coverings had lower vitamin D status than similar women who dressed according to western norms. Additionally, we examined if vitamin D status correlated with muscular strength and bone status in both populations and if differences in vitamin D status were possibly due to differences in clothing, dietary intake or supplement use. 

## 2. Methods

### 2.1. Participant Inclusion Criteria

This study compared hijab-wearing women to pair-matched women who dressed according to western norms and was performed in Halifax, NS, Canada. A sample of 11 women who had dressed in head-to-toe garments for more than a year immediately previous to the study represented the study group. Either partial or full exposure of their hands, face and feet was acceptable. The comparative group was comprised of 11 women who were each pair matched with a study subject and wore westernized styles of dress as defined by willingness to fully expose skin on the arms, legs, face and parts of the torso during summer months, at least occasionally. Women were eligible for the comparative group if they were within ±5 years of age, ±6 cm in height and ±10 kg in weight of a study group participant and had similar skin tone, as judged visually by a researcher. Eligibility criteria for inclusion for all participants were: premenopausal, at least 18 years old, ambulatory, light to olive skin tone, not pregnant and had resided in Nova Scotia continuously for at least a year immediately previous to the study. To ensure these criteria were met, a preliminary screening questionnaire was administered, which summarized medical, skeletal and muscular disease history. Women on medications for diabetes mellitus, intestinal, hepatic, renal, thyroid, muscular or skeletal disorders and those who had used tanning beds within six months or had been being previously immobile for at least three months were excluded from the study. Eligible subjects provided informed consent before participating in the study. The study protocol was approved by the Capital Health District Authority clinical Research Ethics Board (protocol CDHA-RS/2009-275).

### 2.2. Participant Recruitment

Study group participants were recruited by mass emails, flyers, brochures, posters or brief presentations at the Muslim Student Association (MSA) of Dalhousie University, the regional Muslim sisterhoods, inter-faith centres and Immigrant Settlement and Integration Services (ISIS) Halifax. These recruitment strategies led to a snowball effect and via word of mouth other interested women were informed of the research project. Participants in the comparative group were recruited by flyers, posters, and also via word of mouth from interested women to others. 

### 2.3. Study Design

This study used a cross-sectional, matched pair design. Data collection began in February, 2009. As a result of consistently cool and cloudy weather conditions throughout May and June, data collection continued into June. The primary independent variable for this study was concealing *versus* westernized clothing, whereas the primary dependent variable measured was serum vitamin D. Muscular strength and bone status comprised the secondary dependent variables. Additional independent variables evaluated were height, weight, level of weekly physical activity and dietary intakes of calcium, vitamin D and other forms of supplementation. The same tester and instruments were employed throughout the investigation in order to avoid inter-rater and inter-instrumental differences. Confidentiality and anonymity were secured through the use of private rooms and coding of documents and data.

### 2.4. Questionnaires

All prospective volunteers completed a brief preliminary screening questionnaire to identify those who were eligible for the study. Approved participants were asked to complete a more detailed questionnaire, which included questions about fracture history, physical activity, smoking status, medication, dietary habits and sun exposure. Each participant was asked to recall their history, frequency and duration of participation in moderate to vigorous physical exercise such as sports, weight training, or cardiovascular exercise. Each reported activity was categorized as osteogenic or non-osteogenic in accordance with a previous guideline [20]. Exercise categorized as osteogenic also corresponded to land-based moderate to vigorous exercise for cardiovascular effects. Subjects were also questioned as to how long they walked or ran during a typical day and in the past. 

Each subject was interviewed by a trained researcher in order to complete a 24 h recall dietary exercise based on the Automated Multiple-Pass Method [21,22]. Daily dietary intake of fortified milk, cheese, yogurt, juice, margarine and total sodium were also recorded. Each participant was asked to bring with them containers for any medication, supplements or multivitamins that they consumed regularly. Any supplementation and the brand and dosage of all supplements were noted and the approximate amounts of calcium and vitamin D ingested in supplements were estimated. The collected information was entered into a nutritional analysis software program, EatRight^®^ Version 15.0 (Jones & Bartlett Learning, Burlington, MA, USA). 

### 2.5. Serum Vitamin D Analysis

Each participant was informed that the blood draw needed for vitamin D data was optional. Women who volunteered for blood sampling underwent a blood draw by a female phlebotomist. All serum samples were analyzed for 25(OH)D by chemiluminescence immunoassay (CLIA) at the Hospitals In-Common Laboratory Inc., Toronto, Ontario. The precision coefficient of variation for this laboratory is 2.9–5.5% within a run of samples and 6.3–12.9% for total samples.

### 2.6. Bone Status Measurement

Calcaneal bone testing was performed using the Achilles InSight Quantitative Ultrasonometer (QUS) (General Electric, WI, USA). All testing was performed according to the recommendations of the manufacturer. Before each testing session a calibration assessment was performed using a phantom provided by the manufacturer as a measure of quality assurance. Three trials were performed on the right heel of each subject with full repositioning between measurements. From these scans broadband ultrasound attenuation (BUA) and speed of sound (SOS) were measured and stiffness index (SI) was calculated. Previous measurements performed on the same machine in this laboratory demonstrated an *in vivo* precision of SI of 0.4 to 2.2%. 

### 2.7. Muscular Strength Measurement

Muscular strength of each subject was assessed via handgrip strength tests using an A5401 digital hand grip dynamometer (Takei Scientific Instruments Co. Ltd., Tokyo, Japan). With the device fitted to the hand, each participant was required to stand with her feet shoulder width apart and arm lowered but slightly elevated away from the side of the body that was holding the device. Upon instruction the participant inhaled and with exhalation was directed to compress the hand grip device as firmly as possible without touching the body. Two alternating trials, beginning with the left hand, were performed on each hand with a 30 to 60 s rest period between each compression. The averages of the two trials for the left and right hand were calculated separately and then summed to provide a total grip strength value. Hand dominance was also recorded. 

### 2.8. Additional Measurements

The height and weight of each participant was measured using a Detecto double beam physician’s scale with stadiometer (Detecto Scale Co., Webb City, MO, USA). All participants removed their outdoor shoes and jackets but remained fully clothed during height and weight measurement. Body mass index (BMI) was calculated from these measurements.

### 2.9. Statistical Analyses

Prior to statistical analyses, data were subjected to Kolmogorov-Smirnov tests, which confirmed normal distribution of all variables. Descriptive statistics were calculated for most variables and reported as means ± SD. A paired sample *t*-test was used to determine if significant differences existed between groups in mean serum vitamin D, bone status, muscular strength and additional variables from the questionnaire. Independent *t*-tests were also employed to examine differences between groups for these parameters because not all women had blood drawn and therefore larger sample sizes were examined this way. Additionally, differences between groups for s-25(OH)D and grip strength were tested in unpaired data using multivariate analysis of variance (MANOVA) with vitamin D intake as a covariate. Similarly, differences between groups for hand strength were tested in unpaired data using MANOVA with both low intensity and moderate to high intensity exercise as covariates. All 11 matched pairs were used to assess differences between groups in bone status, muscular strength, physical activity and dietary habits. Fisher’s exact tests (FET) were used to determine if significant relationships existed between type of clothing worn, and vitamin D level and other lifestyle and medical variables. Linear regression was used to analyze the relationship between vitamin D and bone status and muscular strength. Statistical significance was accepted at *p* ≤ 0.05 whereas *p*-values between 0.05 and 0.1 indicated a notable trend due to the small sample size. SPSS version 15 was used for all analyses.

## 3. Results

### 3.1. Participants

For this investigation 31 study group women expressed an interest in participating and 22 agreed to complete the screening questionnaire of which 17 met the inclusion criteria and qualified to continue with testing. Eleven of the remaining 17 women who were eligible as study group participants signed the consent form and participated in the bone and muscle testing session. Nine of the final 11 study group participants agreed to blood sampling. 

In total 24 women who dressed according to western norms expressed interest in participating in this investigation and 19 of these completed the screening questionnaire. Three did not meet inclusion criteria, which left 16 women eligible to participate as comparative group subjects. Eleven of the remaining 16 women were determined to be suitable as matched pairs to study group subjects. In total this resulted in a comparative group with 11 participants match paired to each study group subject. However, only seven of the comparative group women agreed to blood sampling. The demographic and anthropometric characteristics of the study and control groups are presented in Table 1. Paired sample t-tests detected no statistical difference between the two groups for age, height or weight. Additional information relevant to bone status or lifestyle behaviour is also included in Table 1. Women in both groups reported at least moderate exposure (1 h, 3 to 4 times per week) to sunlight during peak UVB hours.

**Table 1:** Subject characteristics of women who wore concealing *versus* western-style clothing.

Characteristic	Concealed ( *n =* 11)	Western ( *n =* 11)
Age (year) ^a^	23.2 ± 2.1	25.1 ± 3.3
Height (cm) ^a^	160.0 ± 4.2	160.4 ± 7.1
Weight (kg) ^a^	60.5 ± 7.8	60.5 ± 9.8
BMI (kg·m^−^^2^) ^a^	23.5 ± 2.2	23.4 ± 2.4
Smokers	0	0
History of skeletal fractures	0	5
Hormonal contraceptive use	0	5
Regular sunscreen use	1	7

^a^ Biometric data are means ± SD, none of the means were statistically different (*p* ≤ 0.1). BMI = Body mass index.

### 3.2. Serum 25-Hydroxyvitamin D

**Figure 1:**
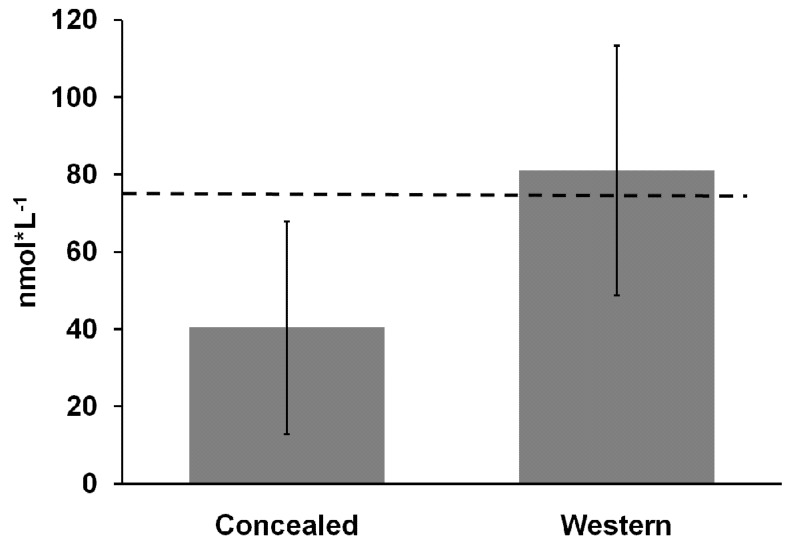
The means ± SD of serum 25-hydroxyvitamin D (s-25(OH)D) levels for women who wore concealing clothing compared to pair matched women who dressed according to western norms. The dashed line represents the level of s-25(OH)D that is considered to be sufficient.

Nine of the 11 study group and seven of the 11 comparative group subjects agreed to participate in the blood draw for 25(OH)D testing. This allowed for seven matched pairs to be statistically analyzed for differences in serum vitamin D levels. Given the controversy in the literature regarding what is an insufficient *versus* deficient level of s-25(OH)D, the levels were defined in this study as follows: severely deficient, <30 nmol·L^−1^; deficient, 30–49 nmol·L^−1^; insufficient, 50–74 nmol·L^−1^; and sufficient, ≥75 nmol·L^−1^ [23]. The mean s-25(OH)D concentration for the seven hijab-wearing women was significantly lower (*p* = 0.01) than that of their matched pairs who wore western-style clothing (Figure 1). When the difference in s-25(OH)D between groups was adjusted for vitamin D intake, s-25(OH)D remained lower in the hijab-wearing group (*p* = 0.05). Although there was considerable variance within each group, every woman in the study group had a lower s-25(OH)D than her matched pair. Additionally, the mean s-25(OH)D for all nine study group participants was lower than the mean of the 7 matched study group women, at 36.0 ± 25.5 nmol·L^−1^. Six of eight study group women had severe vitamin D deficiency (s-25(OH)D < 30 nmol·L^−1^) but no comparative group women did. A trend between clothing type and severe vitamin D deficiency was detected (*p* = 0.06; FET). However, within the subset of participants who had vitamin D levels less than 75 nmol·L^−1^, a significant negative relationship between sunscreen use and s-25(OH)D was detected through linear regression (*p* = 0.03). 

### 3.3. Bone Status and Muscle Strength

The hijab-wearing and non-hijab-wearing groups were very similar in all measures of bone status (Table 2). When the SI value of each of these samples was compared using a one sample *t*-test to those of 125 healthy, local, premenopausal women aged 18 to 25 years whose mean SI was 108.1 ± 16.4, no significant differences were detected between the local sample and the mean SI of the study and comparative groups (study group, *p* = 0.9; comparative group, *p* = 0.8, respectively). 

**Table 2:** Bone and muscle strength of women who wore concealing *versus* western-style clothing.

Measures	Concealed ( *n =* 11)	Western ( *n =* 11)	*p*-value
QUS			
SOS (m·s^−^^1^)	1578.1 ± 11.8	1581.9 ± 8.2	0.8
BUA (dB·MHz^−^^1^)	130.6 ± 5.6	130.6 ± 5.4	0.9
SI	108.8 ± 6.6	109.6 ± 5.5	0.9
Hand grip			
Right hand (kg)	25.0 ± 4.4	29.4 ± 2.0	<0.05
Left hand (kg)	23.8 ± 4.1	26.4 ± 6.5	0.2
Total grip (kg)	48.8 ± 8.0	55.8 ± 12.8	0.09

Data are means ± SD. QUS, quantitative ultrasound; SOS, speed of sound; BUA, broadband ultrasound attenuation; SI, Stiffness Index.

The women who wore concealing clothes displayed 12.5% lower combined hand grip strength than their matched pairs (Table 2). Although this only represented a trend towards lower total hand grip in the hijab-wearing women, the differences were greater in the right hand which was most often the dominant hand, than for the left hand. However, when the hand grip results were adjusted for amount of moderate to high intensity exercise, no differences remained between groups. Instead, total grip, right handed and left handed grip strength were significantly related to moderate to high intensity exercise (*p* = 0.02, 0.03, 0.03, respectively) but not low intensity exercise. 

### 3.4. Nutrient Intake

Dietary intakes from the 24-h dietary recall indicated that the women in the concealing clothing group consumed about half the vitamin D in food and supplements than did the women in the comparative group (Table 3). Differences in macronutrient content were not significant between groups although the hijab-wearing women consumed 22% more total fat than did their western-clothed counterparts. Daily consumption of milk, margarine and fortified juice were not significantly different between the groups. Consumption of multivitamin supplements was reported in two of the study and six of the comparative group participants. Of the 16 participants who agreed to blood sampling, six who did not consume vitamin D supplements presented severe vitamin D deficiency (<30 nmol/L) whereas severe vitamin D deficiency was not observed in those who complemented their diet with multivitamins, fish oils or vitamin D supplementation (*p* = 0.01; FET). Overall, a trend of increased s-25(OH)D and multivitamin or vitamin D supplement use was observed (*p* = 0.1). The relationship between overall dietary vitamin D intake, including supplementation of any kind, and s-25(OH)D concentration was weak (*R*^2^ = 0.2) although present (*p* = 0.07). 

**Table 3:** Nutrient intake by women who wore concealing *versus* western-style clothing.

Nutrient per Day	Concealed ( *n =* 11)	Western ( *n* = 11)	*p*-value
Vitamin D * (IU)	316.3 ± 353.1	601 ± 340.9	0.001
Calcium * (mg)	1515.9 ± 674.8	1528.9 ± 546.5	0.9
Sodium (mg)	2005.3 ± 1309.5	2678.7 ± 652.7	0.1
Energy (kcal)	1823.9 ± 784.3	1883.3 ± 406.7	0.8
Protein (g)	89.9 ± 32.8	80.0 ± 22.0	0.4
Carbohydrate (g)	243.0 ± 108.2	282.2 ± 82.5	0.4
Fat (g)	56.8 ±38.9	46.6 ± 12.4	0.5

Data are means ± SD. * Intake from food plus supplements.

### 3.5. Exercise

The women who wore concealing clothing participated in less moderate to high impact exercise than their counterparts (2.4 h·week^−1^ *vs.* 6.4 h·week^−1^; *p* = 0.05) and had done so for fewer years (1.9 years *vs.* 8.6 years; *p* = 0.03). They had also participated in less low impact exercise than their counterparts (1.1 h·week^−1^*vs.* 2.9 h·week^−1^; *p* = 0.04) although the duration of such activities was not as different between groups (2.5 years *vs.* 3.4 years; *p* = 0.5). A weak (*R*^2^ = 0.26) but significantly positive relationship (*p* = 0.01) was detected between frequency of high impact exercise and muscular strength but not bone status. Neither bone status nor muscular strength was related to frequency or duration of low impact exercise.

## 4. Discussion

To our knowledge this study is the first to report the vitamin D status of Muslim women who wear concealing clothes and reside in Canada and the first to report vitamin D status, dietary vitamin D intakes, bone status and muscle strength in a cohort of Muslim women in a western country. The mean inadequate s-25(OH)D level of 36 nmol·L^−1^ that we report for the nine young hijab-wearing women in Halifax, Nova Scotia is similar to that measured in similarly dressed young women in Bangladesh [24], Iran [25] and Turkey [26]. Given that living year round at lower latitudes provides more opportunity for cutaneous conversion of vitamin D, the even lower vitamin D status of pre-menopausal women reported in Jordan [27], Iran [28], Saudi Arabia [29], Turkey [30] and Lebanon [31] appears to be related to the practice of wearing a hijab and other body coverings. A few studies have reported higher levels of s-25(OH)D in young women in such Islamic countries as Saudi Arabia [32], Morocco [33], and Iran [34] but their levels of s-25(OH)D were still inadequate for optimal health. 

Women who reside in more northern countries and continue to wear full body coverings may be at risk of more severe hypovitaminosis due to the seasonal lack of UVB radiation from the sun. Studies from latitudes higher than Halifax support this hypothesis. Older women from Pakistan who resided in England (53° N) had s-25(OH)D levels of 11 nmol/L compared to ethnic English women who had levels of 47 nmol·L^−1^ [35]. Similarly, Somali women in Finland (60° N) had lower s-25(OH)D levels than ethnic Finns (37 *vs.* 54 nmol·L^−1^) [36] as did Turkish and Moroccan women in the Netherlands (52° N) compared to their ethnic Dutch counterparts (15, 20, *vs.* 53 nmol·L^−1^) [37]. Reports suggest that this problem is also common to locations at latitudes similar to Halifax (43° N). Concealed woman in France (45° N) had s-25(OH)D levels of 20 nmol·L^−1^ compared to 39 nmol·L^−1^ for those who wore European clothing [38] while veiled women in Michigan, USA (42° N) averaged a mere 10 nmol·L^−1^ if they did not take supplements [39]. 

The hijab-wearing women in our study consumed less vitamin D both from food and supplements and this may have contributed to their lower s-25(OH)D levels. Low dietary intake of vitamin D has also been noted in other similar cohorts of women who reside in western countries where vitamin D fortified foods are common [39,40,41]. Far fewer of the hijab-wearing women in our study and in other studies [2,36] consumed vitamin supplements than do secularly dressed women even though such supplements are readily available and quickly raise s-25(OH)D levels [42]. 

The hijab-wearing women in our study exhibited lower hand grip strength than did the western-dressed women although the difference was only detected in the right hand. This positive relationship between a deficiency in hand grip and inadequate vitamin D status was previously reported in women [43]. However, the lower levels of participation in moderate to vigorous exercise habitually performed by the women in this study, rather than vitamin D intake, appeared to result in their lower grip strength. If a deficit in muscular strength is perpetuated into older age, then the ability to perform basic activities of daily living (ADL) can be compromised [44]. Therefore, the benefits of participation in higher intensity exercise for muscular strength should be recommended for all women regardless of dress. The hijab-wearing women in this study were mostly young, well-educated women who participated in sports so their results probably do not represent those of older, less active or more conservatively dressed women for whom a vitamin D deficiency is more likely to correspond with a strength deficit. Therefore, deficits in muscular strength in concealed older women may be warranted.

No differences were found between the bone status of the hijab-wearing and western-clothed women in this study. This is somewhat surprising, given that the levels of vitamin D in some of these women were as low as those who experienced bone pathologies in Iran [28] and Turkey [45]. However, others have reported no bone differences with inadequate vitamin D status in women who wear concealing clothes [33,46]. Possibly the high calcium intake by the hijab-wearing women in Halifax prevented bone deficits to manifest due to inadequate vitamin D synthesis and consumption. The detrimental effect of low s-25(OH)D on bones might also have been mitigated by the substantial amount of moderate to vigorous exercise that the women in this study reported to perform. Although they exercised less than the western-clothed women, this is unlikely to be relevant because very few daily stimuli that exceed a threshold in strain rate are required to initiate mechanotransduction [47]. The substantial difference we report between the hijab-wearing and their western-clothed pairs is partially due to the higher mean s-25(OH)D level of 81 nmol·L^−1^ measured in the western-dressed women. Other Canadian studies have reported 52–64 nmol·L^−1^ for winter and 69–74 nmol·L^−1^ in summer and fall in young Caucasian adults [8,48]. The most comparable women were reported in a study from the adjacent province of Newfoundland, which has a similar climate to Halifax [49]. Their mean values for young women in Newfoundland were somewhat lower than ours, at 69 nmol·L^−1^ in July to August. The reason for the somewhat higher than expect values for the western-clothed women in our study is not clear, especially given that sampling was completed prior to the season of optimal cutaneous synthesis. However, they did consume twice the amount of vitamin D, and more of it from supplements, than did the hijab-wearing women, and supplement use is an effective method to raise s-25(OH)D levels [50]. 

This pilot study has several limitations. The small sample size and lack of inclusion of more conservatively dressed and older hijab-wearing women suggest that these results may not be applicable to other women who wear concealing clothing in Nova Scotia or elsewhere. The self-reported basis of the exercise participation may have contributed to further error in interpreting what factors resulted in low muscular strength. Future studies should use objective measures, such as accelerometry, to determine exercise intensity. Also, seasonal differences in s-25(OH)D are well known and therefore the results of this mostly springtime study might not be applicable to a different season. Additionally, matching the women between groups for skin tone was performed on a visual basis whereas an objective method would be more reproducible. 

## 5. Conclusions

The s-25(OH)D measured in women in coastal Canada who wore concealing clothes was lower than in secularly dressed women and several of these women were severely deficient in vitamin D. Although these women also consumed less vitamin D, their lower s-25(OH)D appeared to be attributable to an additional cause, which could be their attire. Conversely, the lower muscular strength measured in the women who dressed in concealing clothes appeared to be due to lower participation in moderate to vigorous physical exercise rather than to the deficit in vitamin D. This pilot study suggests that vitamin D supplementation is needed by women who wear concealing clothes and live in a northern maritime location.

## Implications

This pilot study suggests that the increasing numbers of women living in northern maritime regions who adhere to a dress code of concealing clothing need to supplement their diets with vitamin D in order to reach or maintain a healthy vitamin D status. 

## References

[1] Madar A.A., Stene L.C., Meyer H.E. (2009). Vitamin D status among immigrant mothers from Pakistan, Turkey and Somalia and their infants attending child health clinics in Norway. Br. J. Nutr..

[2] Andersen R., Molgaard C., Skovgaard L.T., Brot C., Cashman K.D., Jakobsen J., Lamberg-Allardt C., Ovesen L. (2008). Pakistani immigrant children and adults in Denmark have severely low vitamin D status. Eur. J. Clin. Nutr..

[3] Pal B.R., Marshall T., James C., Shaw N.J. (2003). Distribution analysis of vitamin D highlights differences in population subgroups: preliminary observations from a pilot study in UK adults. J. Endocrinol..

[4] Gozdzik A., Barta J.L., Wu H., Wagner D., Cole D.E., Vieth R., Whiting S., Parra E.J. (2008). Low wintertime vitamin D levels in a sample of healthy young adults of diverse ancestry living in the Toronto area: associations with vitamin D intake and skin pigmentation. BMC Public Health.

[5] Vieth R., Cole D.E., Hawker G.A., Trang H.M., Rubin L.A. (2001). Wintertime vitamin D insufficiency is common in young Canadian women, and their vitamin D intake does not prevent it. Eur. J. Clin. Nutr..

[6] Waiters B., Godel J.C., Basu T.K. (1999). Perinatal vitamin D and calcium status of northern Canadian mothers and their newborn infants. J. Am. Coll. Nutr..

[7] Rucker D., Allan J.A., Fick G.H., Hanley D.A. (2002). Vitamin D insufficiency in a population of healthy western Canadians. CMAJ.

[8] Gozdzik A., Barta J.L., Weir A., Cole D.E., Vieth R., Whiting S.J., Parra E.J. (2010). Serum 25-hydroxyvitamin D concentrations fluctuate seasonally in young adults of diverse ancestry living in Toronto. J. Nutr..

[9] Newhook L.A., Sloka S., Grant M., Randell E., Kovacs C.S., Twells L.K. (2009). Vitamin D insufficiency common in newborns, children and pregnant women living in Newfoundland and Labrador, Canada. Matern. Child Nutr..

[10] LeBoff M.S., Kohlmeier L., Hurwitz S., Franklin J., Wright J., Glowacki J. (1999). Occult vitamin D deficiency in postmenopausal US women with acute hip fracture. JAMA.

[11] Heidari B., Shirvani J.S., Firouzjahi A., Heidari P., Hajian-Tilaki K.O. (2010). Association between nonspecific skeletal pain and vitamin D deficiency. Int. J. Rheum. Dis..

[12] Razzaghy-Azar M., Shakiba M. (2010). Assessment of vitamin D status in healthy children and adolescents living in Tehran and its relation to iPTH, gender, weight and height. Ann. Hum. Biol..

[13] Moussavi M., Heidarpour R., Aminorroaya A., Pournaghshband Z., Amini M. (2005). Prevalence of vitamin D deficiency in Isfahani high school students in 2004. Horm. Res..

[14] Hatun S., Islam O., Cizmecioglu F., Kara B., Babaoglu K., Berk F., Gokalp A.S. (2005). Subclinical vitamin D deficiency is increased in adolescent girls who wear concealing clothing. J. Nutr..

[15] Foo L.H., Zhang Q., Zhu K., Ma G., Hu X., Greenfield H., Fraser D.R. (2009). Low vitamin D status has an adverse influence on bone mass, bone turnover, and muscle strength in Chinese adolescent girls. J. Nutr..

[16] Wang Y., DeLuca H.F. (2011). Is the vitamin d receptor found in muscle?. Endocrinology.

[17] de Torrente de la Jara G., Pecoud A., Favrat B. (2004). Musculoskeletal pain in female asylum seekers and hypovitaminosis D3. BMJ.

[18] de Torrente de la Jara G., Pecoud A., Favrat B. (2006). Female asylum seekers with musculoskeletal pain: the importance of diagnosis and treatment of hypovitaminosis D. BMC Fam. Pract..

[19] Glerup H., Mikkelsen K., Poulsen L., Hass E., Overbeck S., Andersen H., Charles P., Eriksen E.F. (2000). Hypovitaminosis D myopathy without biochemical signs of osteomalacic bone involvement. Calcif. Tissue Int..

[20] Welch J.M., Rosen C.J. (2005). Older women track and field athletes have enhanced calcaneal stiffness. Osteoporos. Int..

[21] Blanton C.A., Moshfegh A.J., Baer D.J., Kretsch M.J. (2006). The USDA Automated Multiple-Pass Method accurately estimates group total energy and nutrient intake. J. Nutr..

[22] Moshfegh A.J., Rhodes D.G., Baer D.J., Murayi T., Clemens J.C., Rumpler W.V., Paul D.R., Sebastian R.S., Kuczynski K.J., Ingwersen L.A. (2008). The US Department of Agriculture Automated Multiple-Pass Method reduces bias in the collection of energy intakes. Am. J. Clin. Nutr..

[23] Holick M.F. (2007). Vitamin D deficiency. N. Engl. J. Med..

[24] Islam M.Z., Shamim A.A., Kemi V., Nevanlinna A., Akhtaruzzaman M., Laaksonen M., Jehan A.H., Jahan K., Khan H.U., Lamberg-Allardt C. (2008). Vitamin D deficiency and low bone status in adult female garment factory workers in Bangladesh. Br. J. Nutr..

[25] Mirsaeid Ghazi A.A., Rais Zadeh F., Pezeshk P., Azizi F. (2004). Seasonal variation of serum 25 hydroxy D3 in residents of Tehran. J. Endocrinol. Invest..

[26] Guzel R., Kozanoglu E., Guler-Uysal F., Soyupak S., Sarpel T. (2001). Vitamin D status and bone mineral density of veiled and unveiled Turkish women. J. Womens Health Gend. Based Med..

[27] Mallah E., Hamad M., ElManaseer M., Qinna N., Idkaidek N., Arafat T., Matalka K. (2011). Plasma concentrations of 25-hydroxyvitamin D among Jordanians: Effect of biological and habitual factors on vitamin D status. BMC Clin. Pathol..

[28] Kazemi A., Sharifi F., Jafari N., Mousavinasab N. (2009). High prevalence of vitamin D deficiency among pregnant women and their newborns in an Iranian population. J. Womens Health (Larchmt.).

[29] Al-Daghri N.M., Al-Attas O.S., Al-Okail M.S., Alkharfy K.M., Al-Yousef M.A., Nadhrah H.M., Sabico S.B., Chrousos G.P. (2010). Severe hypovitaminosis D is widespread and more common in non-diabetics than diabetics in Saudi adults. Saudi Med. J..

[30] Alagol F., Shihadeh Y., Boztepe H., Tanakol R., Yarman S., Azizlerli H., Sandalci O. (2000). Sunlight exposure and vitamin D deficiency in Turkish women. J. Endocrinol. Invest..

[31] Gannage-Yared M.H., Chemali R., Yaacoub N., Halaby G. (2000). Hypovitaminosis D in a sunny country: Relation to lifestyle and bone markers. J. Bone Miner. Res..

[32] Ardawi M.-S., Qari M., Rouzi A., Maimani A., Raddadi R. (2011). Vitamin D status in relation to obesity, bone mineral density, bone turnover markers and vitamin D receptor genotypes in healthy Saudi pre- and postmenopausal women. Osteoporos. Int..

[33] Allali F., El Aichaoui S., Khazani H., Benyahia B., Saoud B., El Kabbaj S., Bahiri R., Abouqal R., Hajjaj-Hassouni N. (2009). High prevalence of hypovitaminosis D in Morocco: Relationship to lifestyle, physical performance, bone markers, and bone mineral densiity. Semin. Arthritis Rheum..

[34] Hovsepian S., Amini M., Aminorroaya A., Amini P., Iraj B. (2011). Prevalence of vitamin D deficiency among adult population of Isfahan City, Iran. J. Health Popul. Nutr..

[35] Lowe N.M., Mitra S.R., Foster P.C., Bhojani I., McCann J.F. (2010). Vitamin D status and markers of bone turnover in Caucasian and South Asian postmenopausal women living in the UK. Br. J. Nutr..

[36] Islam M.Z., Viljakainen H.T., Karkkainen M.U., Saarnio E., Laitinen K., Lamberg-Allardt C. (2011). Prevalence of vitamin D deficiency and secondary hyperparathyroidism during winter in pre-menopausal Bangladeshi and Somali immigrant and ethnic Finnish women: Associations with forearm bone mineral density. Br. J. Nutr..

[37] van der Meer I.M., Karamali N.S., Boeke A.J., Lips P., Middelkoop B.J., Verhoeven I., Wuister J.D. (2006). High prevalence of vitamin D deficiency in pregnant non-Western women in The Hague, Netherlands. Am. J. Clin. Nutr..

[38] Le Goaziou M.F., Contardo G., Dupraz C., Martin A., Laville M., Schott-Pethelaz A.M. (2011). Risk factors for vitamin D deficiency in women aged 20-50 years consulting in general practice: A cross-sectional study. Eur. J. Gen. Pract..

[39] Hobbs R.D., Habib Z., Alromaihi D., Idi L., Parikh N., Blocki F., Rao D.S. (2009). Severe vitamin D deficiency in Arab-American women living in Dearborn, Michigan. Endocr. Pract..

[40] Reed S.D., Laya M.B., Melville J., Ismail S.Y., Mitchell C.M., Ackerman D.R.  (2007). Prevalence of vitamin D insufficiency and clinical associations among veiled East African women in Washington State. J. Womens Health (Larchmt.).

[41] Holvik K., Meyer H.E., Haug E., Brunvand L. (2005). Prevalence and predictors of vitamin D deficiency in five immigrant groups living in Oslo, Norway: The Oslo Immigrant Health Study. Eur. J. Clin. Nutr..

[42] Islam M.Z., Shamim A.A., Viljakainen H.T., Akhtaruzzaman M., Jehan A.H., Khan H.U., Al-Arif F.A., Lamberg-Allardt C. (2010). Effect of vitamin D, calcium and multiple micronutrient supplementation on vitamin D and bone status in Bangladeshi premenopausal garment factory workers with hypovitaminosis D: A double-blinded, randomised, placebo-controlled 1-year intervention. Br. J. Nutr..

[43] Gupta R., Sharma U., Gupta N., Kalaivani M., Singh U., Guleria R., Jagannathan N.R., Goswami R. (2010). Effect of cholecalciferol and calcium supplementation on muscle strength and energy metabolism in vitamin D-deficient Asian Indians: A randomized, controlled trial. Clin. Endocrinol. (Oxf.).

[44] Hasegawa R., Islam M.M., Lee S.C., Koizumi D., Rogers M.E., Takeshima N. (2008). Threshold of lower body muscular strength necessary to perform ADL independently in community-dwelling older adults. Clin. Rehabil..

[45] Hayirlioglu D.A., Gokaslan H., Cimsit C., Serin N.O. (2008). The impact of clothing style on bone mineral density among women in Turkey. Rheumatol. Int..

[46] Guler T., Sivas F., Baskan B.M., Gunesen O., Alemdaroglu E., Ozoran K. (2007). The effect of outfitting style on bone mineral density. Rheumatol. Int..

[47] Umemura Y., Ishiko T., Tsujimoto H., Miura H., Mokushi N., Suzuki H. (1995). Effects of jump training on bone hypertrophy in young and old rats. Int. J. Sports Med..

[48] Langlois K., Greene-Finestone L., Little J., Hidiroglou N., Whiting S. (2010). Vitamin D status of Canadians as measured in the 2007 to 2009 Canadian Health Measures Survey. Health Rep..

[49] Sloka S., Stokes J., Randell E., Newhook L.A. (2009). Seasonal variation of maternal serum vitamin D in Newfoundland and Labrador. J. Obstet. Gynaecol. Can..

[50] Whiting S.J., Langlois K.A., Vatanparast H., Greene-Finestone L.S. (2011). The vitamin D status of Canadians relative to the 2011 Dietary Reference Intakes: an examination in children and adults with and without supplement use. Am. J. Clin. Nutr..

